# Ultrasonographic evaluation of diaphragm function in patients with chronic obstructive pulmonary disease: A systematic review and meta-analysis

**DOI:** 10.1097/MD.0000000000032560

**Published:** 2022-12-23

**Authors:** Zeng Hua-Rong, Chen Liang, Liu Rong, Tu Yi-Fan, Shi Dou-Zi, Chen Yue, Liu Zu-Lin

**Affiliations:** a The First College of Clinical Medical Science, China Three Gorges University & Ultrasound Department of Yichang Central People’s Hospital, Yichang, Hubei, China; b Yichang Maternal and Child Health Hospital, Yichang, Hubei, China.

**Keywords:** chronic obstructive pulmonary disease, diaphragm, meta-analysis, systematic review, ultrasound

## Abstract

Some studies have reported using ultrasonic evaluations to assess diaphragm function in patients with chronic obstructive pulmonary disease (COPD). However, they have limitations and thus cannot provide strong evidence to support ultrasound evaluations for diaphragm function and dysfunction severity assessments in this patient population. Additionally, quantitative studies on the relationship between ultrasound evaluations and diaphragm function do not exist. Therefore, we performed a systematic review and meta-analysis to explore the usefulness of ultrasonography for evaluating diaphragm function in patients with COPD.

**Methods:**

The Cochrane Library, PubMed, Embase, Web of Science, Chinese Biomedical Literature Database, Wanfang Data, China National Knowledge Network, and Chinese Scientific Journal Database (i.e., VIP) databases were searched for literature about ultrasonic evaluations of diaphragm function in patients with COPD for systematic review. We extracted patient demographic, diaphragm mobility, diaphragm thickness, diaphragm thickening score, and other related parameter data using RevMan 5.3 software for the meta-analysis.

**Results:**

We included 13 articles in the systematic review, 8 of which (494 participants) were included in the meta-analysis. The degree of diaphragm offset in patients with COPD was significantly lower than that in healthy controls (weighted mean difference [WMD] = –1.34; 95% confidence interval [CI]: –2.15, 0.53; *P < *.05). The diaphragm deviation was lower in the severe COPD group than in the mild-to-moderate COPD group (WMD = 0.50; 95% CI: –0.01, 1.01; *P = *.06), but the difference was not significant.

**Conclusion:**

Ultrasonography effectively evaluates diaphragm function in patients with COPD. The diaphragm offset can be used as an auxiliary diagnostic index for COPD, which is also related to disease severity.

## 1. Introduction

The global prevalence of chronic obstructive pulmonary disease (COPD) is 11.7%.^[1]^ COPD is characterized by persistent airflow limitations caused by small airway lesions and pulmonary parenchyma destruction,^[2]^ potentially involving multiple systems. Diaphragm dysfunction is one of the leading causes of mortality in patients with COPD.^[3]^ Currently, X-ray, phrenic nerve stimulation, transdiaphragmatic pressure (Pdi), and electromyography are used to evaluate diaphragm function,^[4–6]^ but each has limitations, including radiation exposure, invasiveness, and technical challenges.

Ultrasound is a rapid, simple, noninvasive, easy-to-perform, repeatable, inexpensive, and radiation-free examination technique used to diagnose and evaluate the severity of lung diseases in critical care medicine since the early 1990s. Currently, diaphragmatic ultrasound is increasingly used to assess patients with COPD. As a noninvasive examination method, ultrasound evaluates diaphragm function by measuring diaphragm thickness, the thickening fraction, and the diaphragm offset.

Several studies have shown that patients with COPD have a significantly lower diaphragm deviation than healthy participants, and the more severe the COPD, the lower the diaphragm offset.^[7–10]^ Furthermore, patients with COPD have thinner diaphragms and lower diaphragm thickening scores than healthy participants.^[11]^

The published studies using ultrasound evaluations to assess diaphragm function in patients with COPD have been single-center studies with a small number of patients. Thus, these isolated results cannot provide strong evidence to support ultrasound evaluations for diaphragm function and dysfunction severity assessments in patients with COPD. In addition, specific quantitative studies on the relationship between ultrasound evaluations and diaphragm function do not exist. Therefore, a comprehensive analysis is urgently needed.

This study systematically reviewed the literature on ultrasound evaluations of diaphragm function in patients with COPD to explore its role in evaluating diaphragm function in this patient population.

## 2. Data and Methods

### 2.1. Inclusion and exclusion criteria

#### 2.1.1. Inclusion criteria.

Study type: case-control or prospective study published in a peer-reviewed journal; Study population: case group: patients diagnosed with COPD based on the Global Initiative for Chronic Obstructive Pulmonary Disease (GOLD) Guidelines issued by the World Health Organization^[12]^ and control group: healthy individuals without recent respiratory disease matched for height, weight, and age during the same period. Interventions: diaphragmatic excursion (cm), diaphragm thickness (cm), and fractional thickening of the diaphragm (%) were measured using ultrasonography in all subjects.

#### 2.1.2. Exclusion criteria.

The exclusion criteria were: repeated publications; abstracts, letters, editorials, expert opinions, reviews, and case reports; studies with missing baseline characteristic data, incomplete data, or the original data in the literature could not be used for the meta-analysis; patients with thoracic deformities or a history of thoracic and abdominal surgery, those who used drugs that affect muscle function, and those with pleural effusion, malignant tumors, or metabolic diseases.

### 2.2. Literature search strategy

We performed electronic searches for relevant literature in the PubMed, Embase, Web of Science, Chinese Biomedical Literature Database, Wanfang Data, China National Knowledge Infrastructure, and Chinese Scientific Journal Database (i.e., VIP) databases. Additionally, the references of all retrieved articles were reviewed for case-control and prospective studies that used ultrasound to evaluate diaphragmatic function in patients with COPD. The English search keywords were: Ultrasonography; Pulmonary disease, chronic obstructive; COPD; and Diaphragm. The Chinese search keywords were: COPD; B-ultrasound; Ultrasound; and Diaphragm.

### 2.3. Literature screening and data extraction

Two researchers with professional knowledge and training independently screened the literature and data table formulations based on the inclusion and exclusion criteria and the Priority Reporting for Systematic Review Meta-Analysis guidelines. Differing opinions were resolved through negotiations.

From the included studies, the reviewers extracted the authors’ names, publication year, study design, sample size, the mean age of study participants (year), ultrasound mode, measurement location, and measurement parameters (including the diaphragm excursion [cm], diaphragm thickness [cm], and diaphragmatic thickening fraction [%]), and comparisons with other methods.

### 2.4. Risk of bias assessment

Two researchers independently evaluated and cross-checked the methodological quality of the literature. Disagreements were resolved through negotiation or consultation with a third researcher. The final included literature was based on the Newcastle-Ottawa Scale table score.^[13]^

### 2.5. Statistical methods

The meta-analysis was performed using RevMan 5.3 software. All data extracted in this study were measurement data, expressed as the weighted mean difference (WMD) and its 95% confidence interval (CI). *P* values of* < *.05 were considered statistically significant. A heterogeneity (*I*^2^) test was performed on the included literature. An *I*^2^ value of ≤ 50% indicated the heterogeneity among the studies was small, and a fixed-effect model was used. An *I*^2^ value of > 50% indicated heterogeneity among the studies, and a random-effects model was used.

## 3. Results

### 3.1. Search results

**Figure 1.**
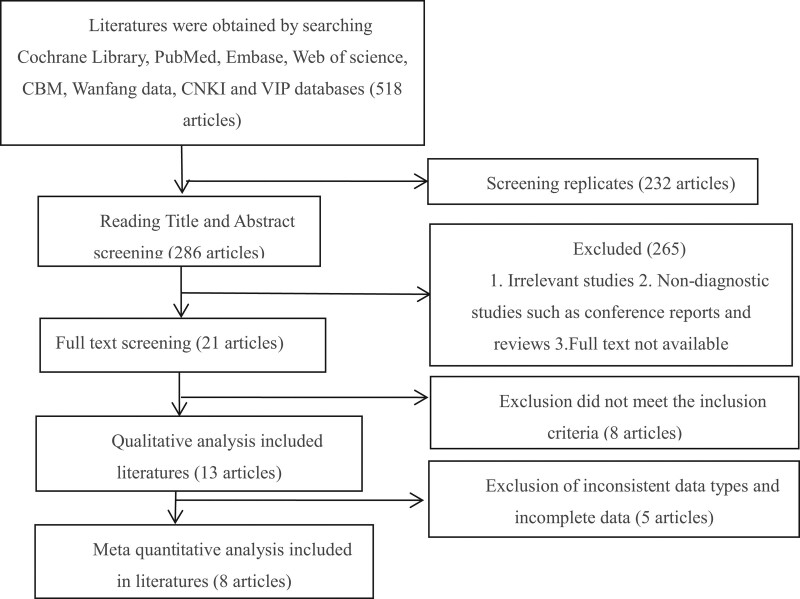
Document retrieval flow chart.

We identified 518 relevant articles in the Chinese and English databases and other literature sources; 13 ^9-21^were included in the systematic review, of which 8 were included in the quantitative analysis. The total sample size for the systematic review was 866; the quantitative meta-analysis included 494 participants: 340 COPD cases, 154 healthy controls, 441 patients who underwent diaphragm excursion assessments, 133 patients who underwent diaphragm thickness assessments, and 184 patients who underwent fractional diaphragmatic thickening assessments. Figure 1 presents a detailed flowchart of the literature search.

### 3.2. Baseline characteristics

Table 1 presents the baseline characteristics of the studies included in this systematic review; 12 were case-control studies, and 1 was a prospective study. The ultrasound measurement mode was the B/M mode, the measurement location was the liver as the acoustic window, and the measurement was performed on the right diaphragm. The study participants were patients diagnosed with COPD based on the GOLD guidelines and healthy volunteers without recent respiratory disease matched for height, weight, and age during the same period.

**Table 1:** Baseline characteristics of the included literature.

First author	Yr of publication	Research and design	Sample size	Age (yrs)	Ultrasonic mode	Measuring position	Measuring parameters	Compare
Ma Ying^[7]^	2018	Case-control study	Case 68 Control 68	52.2 ± 9.7 50.3 ± 9.0	M mode	The right side of the diaphragm	Diaphragm deviation	Pulmonary function
Huang Qiuxia^[8]^	2019	Case-control study	mild-to-moderate 34 severe 30	75.1 ± 7.4 74.7 ± 8.9	B/M mode	The right side of the diaphragm	Diaphragm deviation Diaphragm thickening fraction	Pulmonary function
Wang Jianyao^[9]^	2020	Case-control study	mild-to-moderate 27 severe 11	67.5 ± 5.7	B/M mode	The right side of the diaphragm	Diaphragm deviation Diaphragm thickness	None
Wang Li^[10]^	2019	Case-control study	Case 57 Control 10	79. 7 ± 3. 4 79. 7 ± 3. 7	B/M mode	The right side of the diaphragm	Diaphragm deviation Diaphragm thickening fraction	Percutaneous diaphragm electromyography
Nuttapol Rittayamai^[14]^	2020	Case-control study	Case 80 Control 20	72.0 [64–76] 71.0 [65–79]	B/M mode	The right side of the diaphragm	Diaphragm deviation Diaphragm thickening fraction	Pulmonary function
Nadine Scheibe^[15]^	2015	Case-control study	Case 60 Control 20	/	M mode	The right side of the diaphragm	Diaphragm deviation	Pulmonary function
SungYoon Lim^[11]^	2019	Prospective study	Case 10	79. 8 ± 8.1	B/M mode	The right side of the diaphragm	Diaphragm deviation Diaphragm thickening fraction	Pulmonary function
Li He^[16]^	2014	Case-control study	Case 60 Control 21	66.5 ± 9.4 62.1 ± 7.3	M mode	The right side of the diaphragm	Diaphragm deviation	Pulmonary function
Togay Evrin^[17]^	2019	Case-control study	Case 61 Control 40	71.0 ± 13.0 67.5 ± 10.0	M mode	The right side of the diaphragm	Diaphragm deviation	Pulmonary function
Kazuki Okura^[18]^	2020	Case-control study	Case 38 Control 15	72.0 ± 8.0 75.0 ± 5.0	B mode	The right side of the diaphragm	Diaphragm thickness Diaphragm thickening fraction	Pulmonary function
Priya Ramachandran^[19]^	2020	Case-control study	Case 24 Control 18	61.5 ± 8.4 61.9 ± 7.9	B/M mode	The right side of the diaphragm	Diaphragm deviation Diaphragm thickness	Pulmonary function
Behrooz Davach^[20]^	2014	Case-control study	Case 25 Control 25	59.2 ± 12.0 58.2 ± 11.2	M mode	The right side of the diaphragm	Diaphragm deviation	Pulmonary function
Mahvish Qaiser^[21]^	2020	Case-control study	Case 26 Control 18	62.6 ± 7.5 55.5 ± 9.6	M mode	The right side of the diaphragm	Diaphragm deviation	Pulmonary function

### 3.3. Risk of bias assessment

**Figure 2.**
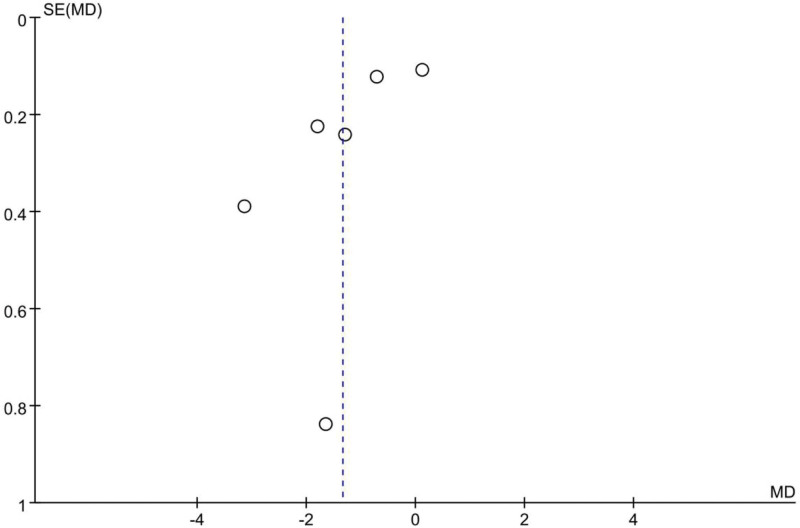
Funnel plot of the diaphragm offset in patients with chronic obstructive pulmonary disease (COPD) and healthy controls.

Table 2 presents the Newcastle-Ottawa Scale scores of the included studies (n = 13), and Figure 2 (funnel plot) illustrates the publication bias analysis results performed on the ultrasound-measured diaphragmatic excursion of patients with COPD and healthy controls.

**Table 2:** Risk bias evaluations (scores).

First author	Yr of publicat-ion	Select object				Compa- rability	Exposure factors			Total score
		Selection of case	Represent-ativeness of cases	Selection of control	Determi-nation of control		Determina-tion of exposure factors	Using the same method	No response rate	
Ma Ying^[7]^	2018	1	1	1	1	2	1	1	1	9
Huang Qiuxia^[8]^	2019	1	1	0	0	2	1	1	1	7
Wang Jianyao^[9]^	2020	1	1	0	0	2	1	1	1	7
Wang Li^[10]^	2019	1	1	1	1	2	1	1	1	9
Nuttapol Rittayamai^[14]^	2020	1	1	1	1	2	1	1	1	9
Nadine scheibe^[15]^	2015	1	1	1	1	2	1	1	1	9
Sung Yoon Lim^[11]^	2019	1	1	0	0	0	1	0	0	3
Li He^[16]^	2014	1	1	1	1	2	1	1	1	9
Togay Evrin^[17]^	2019	1	1	1	1	2	1	1	1	9
Kazuki Okura^[18]^	2020	1	1	1	1	2	1	1	1	9
Priya Ramachandran^[19]^	2020	1	1	1	1	2	1	1	1	9
Behrooz Davach^[20]^	2014	1	1	1	1	2	1	1	1	9
Mahvish Qaiser^[21]^	2020	1	1	1	1	2	1	1	1	9

### 3.4. Qualitative analysis

Of the 13 included studies, 12^[7–11,14–17,19–21]^ measured diaphragmatic excursion, of which 9^[7,10,14–17,19–21]^ found that during deep breathing, diaphragm excursion was significantly lower in the COPD group than in the healthy control group. Furthermore, 7 studies^[8,9,14–17,20]^ classified patients with COPD into mild-moderate and severe cases based on the GOLD classification. They also compared diaphragm displacement between moderate and severe COPD groups. The results demonstrated that diaphragm movement was significantly higher in the mild-to-moderate group than in the severe group.

Additionally, 7 studies^[8–11,14,18,19]^ measured diaphragm thickness and calculated fractional diaphragmatic thickening, reporting that patients with COPD had significantly lower diaphragmatic thickness and fractional diaphragmatic thickening values than the healthy controls, with increased lung hyperinflation. These results indicate an increase in COPD severity and a progressive decrease in the diaphragm thickness. Thus, the diaphragm function of patients with COPD, especially those with severe COPD, was significantly lower than that of healthy controls.

Moreover, the diaphragm and lung functions of patients with COPD were lower than those of healthy controls. Eleven studies^[7,8,11,14–21]^ analyzed the correlation between diaphragm mobility and function indexes, showing that diaphragm mobility positively correlated with pulmonary function parameters, forced expiratory volume in the first second (FEV1), and the FEV1/forced vital capacity ratio in patients with COPD.

### 3.5. Main outcome indicator meta-analysis results

Diaphragm deviation comparisons between the COPD and healthy control groups.

**Figure 3.**
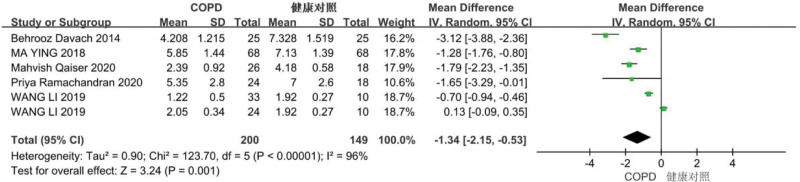
Diaphragm deviation in patients with chronic obstructive pulmonary disease (COPD) and healthy controls.

Six studies were included in this analysis. Heterogeneity among the studies was detected (*P* < .001, *I*^2^ = 96%), and thus the random-effects model was used. We identified a significant difference between the COPD and healthy control groups (WMD = –1.34; 95% CI: –2.15, –0.53, *P = *.001), indicating that the diaphragm deviation degree was significantly lower in patients with COPD than in healthy controls (Fig. 3, Supplemental Digital Content Attachment 1, http://links.lww.com/MD/I254).

Diaphragm offset comparisons among patients with different COPD severities.

**Figure 4.**
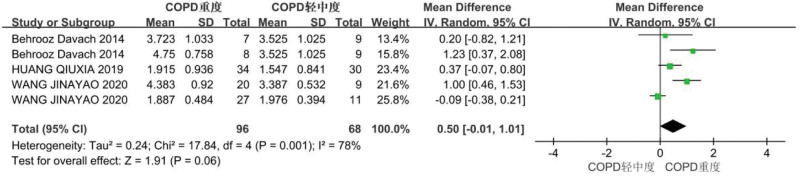
Diaphragm deviation in the mild-to-moderate and severe chronic obstructive pulmonary disease (COPD) groups.

A random-effects model was used to summarize the effect, showing that the diaphragm offset in the severe COPD group was lower than that in the mild-to-moderate COPD group (*P *= .001, *I*^2 ^= 78%), but the difference was not significant (WMD = 0.50, 95% CI: –0.01, 1.01; *P *= .06). This result indicates that the more severe the COPD, the lower the diaphragm offset and the worse the diaphragm function (Fig. 4, Supplemental Digital Content Attachment 2, http://links.lww.com/MD/I255).

### 3.6. Patient and public involvement

No patient or public will be involved.

### 3.7. Ethics and dissemination

Because this study is a secondary analysis, it does not need to be ethically reviewed. The results of this study will be disseminated through peer-reviewed publications, journals, and academic exchanges.

## 4. Discussion

COPD is a chronic disease characterized by airway obstruction and emphysema. The main clinical manifestations are a progressive decline in lung function and a significant decline in patients’ quality of life. For those with COPD, long-term airflow limitation, lung hyperinflation, and morphological changes of the diaphragm, such as flatness, shortening, and a reduction of the involutional area, are closely related to diaphragm dysfunction.^[22]^ The diaphragm is a dome-shaped fibrous muscle between the chest and the abdominal cavity that undertakes approximately 75% of respiratory exercise. Thus, diaphragmatic movements play an important role in breathing. Furthermore, a decreased diaphragm offset or delayed diaphragm movement during deep breathing indicates a functional decline of the diaphragm.^[23]^

Moreover, the diaphragm is an important respiratory muscle for maintaining respiratory ventilation. The mechanical effects caused by systemic inflammation and hyperinflation can lead to diaphragmatic dysfunction in patients with COPD. In addition, oxidative stress, muscle loss, decreased protein production, and increased apoptosis can also lead to diaphragm dysfunction.^[24,25]^ At present, Pdi is the gold standard for measuring diaphragmatic function, but it is invasive and requires patient cooperation, limiting its clinical application. Previous studies have reported a significant correlation between diaphragm offset and Pdi (R^2 ^= 0.33, *P < *.001)^[26]^ and a moderate correlation between diaphragm thickening and Pdi (R^2 ^= 0.40, *P < *.001).^[27]^ Conversely, ultrasound provides a repeatable, noninvasive, and real-time monitoring method for evaluating diaphragm function, and diaphragm offset can be used as an alternative index for measuring diaphragm function.

This study included 13 articles, 12 of which measured the diaphragm offset. The meta-analysis showed that the diaphragm offset in the COPD group was significantly lower than that in the healthy control group (MD = –1.34; 95% CI: –2.15, –0.53; *P = *.001. Also, the diaphragm offset in the severe COPD group was lower than that in the mild-to-moderate COPD group (MD = 0.50, 95% CI: –0.01, 1.01; *P = *.06). A decrease in diaphragm offset indicates that the contractility of the diaphragm decreases in patients with COPD, and the worse the degree of COPD, the lower the contractility of the diaphragm. Decreased contractility is caused by airway obstruction and lung air retention due to bronchitis and emphysema. The diaphragm moves to the caudal side when inhaling and to the cranial side when exhaling. Therefore, COPD can cause lung overinflation, moving the diaphragm to the caudal side, resulting in mechanical defects of the diaphragm and a reduction in contractile force. Previous studies have shown that decreased diaphragm mobility is related to the degree of dyspnea.^[28]^ Therefore, ultrasonic measurement of diaphragm offset is a potential index for diagnosing COPD.

The correlation analysis between diaphragm offset and pulmonary function indices in 11 studies identified a positive correlation between diaphragm mobility and the pulmonary function parameters FEV1 and FEV1/forced vital capacity in patients with COPD. Progress in COPD diaphragm fibers shortens the length of the resting diaphragm, resulting in a decrease in respiratory volume and lung function.^[29]^ In severe COPD or acute exacerbation, patients may not be able to cooperate with pulmonary function tests. However, diaphragm offset measured by ultrasound is simple and easy and can be used as an alternative index to evaluate the risk of COPD deterioration.

Carrillo-Esper et al^[25]^ reported a standardized value of diaphragm thickness in healthy young adults at rest; the average diaphragm thickness in men was 1.9 mm (95% CI: 1.7, 2.0 mm). Furthermore, Kazuki Okura et al demonstrated that the diaphragm thickness and diaphragm thickening fraction of patients with COPD were significantly lower than those of healthy participants, which may be related to diaphragm fiber injury and decreased contractility. Diaphragm ultrasound has become an important evaluation tool for critical monitoring, and diaphragm thickness and thickening fraction have become predictors of successful weaning in mechanically ventilated patients.^[30,31]^ Therefore, diaphragm ultrasonography may be more comprehensive for evaluating diaphragm function in patients with COPD.

Ultrasonic shear wave elastography evaluates diaphragm function by measuring the diaphragm’s elastic shear wave velocity (SWE). One study reported that the diaphragm SWE of patients with COPD at rest at the end of the expiratory period was significantly higher than that of healthy controls. Additionally, diaphragm SWE negatively correlated with pulmonary ventilation function and body mass indexes but positively correlated with health status.^[32]^ Thus, diaphragm measurements may help diagnose, treat, and follow-up assessments of patients with COPD. Also, ultrasound examinations combined with the degree of diaphragm stiffness provide a more comprehensive evaluation of diaphragm function.

There are some limitations in this study, such as the small number of included studies, high heterogeneity among the studies, inconsistent experience of ultrasound examiners, and other factors that potentially produced biases.

## 5. Conclusion

The results of systematic evaluation and meta-analysis show that ultrasonography effectively evaluates the diaphragm function of patients with COPD. Furthermore, the diaphragm offset and thickening fraction of patients with COPD are lower than those of healthy controls and negatively correlate with disease severity.

## Author contributions

**Conceptualization:** Zeng Hua-Rong, Liu Rong.

**Data curation:** Zeng Hua-Rong, Tu Yi-Fan.

**Formal analysis:** Zeng Hua-Rong.

**Funding acquisition:** Liu Rong.

**Investigation:** Zeng Hua-Rong, Chen Liang.

**Methodology:** Zeng Hua-Rong, Tu Yi-Fan.

**Project administration:** Zeng Hua-Rong.

**Resources:** Zeng Hua-Rong, Shi Dou-Zi.

**Software:** Zeng Hua-Rong, Shi Dou-Zi, Liu Zu-Lin.

**Supervision:** Zeng Hua-Rong.

**Validation:** Zeng Hua-Rong.

**Visualization:** Zeng Hua-Rong.

**Writing – original draft:** Zeng Hua-Rong.

**Writing – review & editing:** Zeng Hua-Rong, Liu Rong, Shi Dou-Zi, Chen Yue.

## Supplementary Material
