# Supplementary material for: Ultrasonographic evaluation of diaphragm function in patients with chronic obstructive pulmonary disease: A systematic review and meta-analysis
Source: Medicine (Baltimore). 2022 Dec 23;101(51):e32560. doi: 10.1097/MD.0000000000032560 (PMC9794219; doi:10.1097/MD.0000000000032560)
Supplement: Supplementary file 1 [file medi-101-e32560-s001.pdf]

Attachment1：Diaphragm mobility in healthy control group and COPD group(cm)

Author	Year	COPD			Healthy Control			*P*
		Mean(cm)	SD	Sample	Mean(cm)	SD	Sample	
MAYing	2018	5.850	1.440	68	7.130	1.390	68	＜0.001
WANGLi	2019	1.220	0.500	33	1.920	0.270	10	＜0.001
WANGLi	2019	2.050	0.340	24	1.920	0.270	10	＜0.001
Priya Ramachandran	2020	5.350	2.800	24	7.000	2.600	18	0.07
Behrooz Davach	2014	4.208	1.215	25	7.328	1.519	25	0.024
Mahvish Qaiser	2020	2.390	0.920	26	4.180	0.580	18	＜0.001
