# Supplementary material for: Ultrasonographic evaluation of diaphragm function in patients with chronic obstructive pulmonary disease: A systematic review and meta-analysis
Source: Medicine (Baltimore). 2022 Dec 23;101(51):e32560. doi: 10.1097/MD.0000000000032560 (PMC9794219; doi:10.1097/MD.0000000000032560)
Supplement: Supplementary file 2 [file medi-101-e32560-s002.pdf]

Attachment2：Diaphragm mobility of different degrees of COPD(cm)

Author	Year	Mild to moderate COPD			Severe COPD			*P*
		Mean(cm)	SD	Sample	Mean(cm)	SD	Sample	
HUANGQiuxia	2019	1.915	0.936	34	1.547	0.841	30	0.11
WANGJianyao	2020	1.887	0.484	27	1.976	0.394	11	0.05
WANGJianyao	2020	4.383	0.920	20	3.387	0.532	9	0.06
Behrooz Davach	2014	4.750	0.758	8	3.525	1.025	9	0.024
Behrooz Davach	2014	3.723	1.033	7	3.525	1.025	9	0.024
